# Morphological, phylogenetic, and genomic evidence reveals the causal agent of thread blight disease of cacao in Peru is a new species of *Marasmius* in the section *Neosessiles*, *Marasmius infestans* sp. nov.

**DOI:** 10.12688/f1000research.140405.2

**Published:** 2024-01-29

**Authors:** Angel Fernando Huamán-Pilco, Tito Ademir Ramos-Carrasco, Mario Emilio Ernesto Franco, Daniel Tineo-Flores, Richard Estrada-Cañari, Pedro Eduardo Romero, Vilma Aguilar-Rafael, Lourdes Adriana Ramírez-Orrego, Rosalina Tincopa-Marca, Fanny-Rosario Márquez, Manuel Oliva-Cruz, Jorge Ronny Díaz-Valderrama

**Affiliations:** 1 Grupo de Investigación en Fitopatología y Micología, Instituto de Investigación para el Desarrollo Sustentable de Ceja de Selva, National University Toribio Rodriguez de Mendoza of Amazonas, Chachapoyas, Amazonas, 01001, Peru; 2 Sustainable Plant Protection Programme, Institute of Agrifood Research and Technology (IRTA), 25198 Lieda, Spain; 3 Department of Soil, Plant and Food Sciences, Universita degli Studi di Bari Aldo Moro, Bari, Apulia, 70126, Italy; 4 Centro Experimental Yanayacu, Dirección de Supervisión y Monitoreo en las Estaciones Experimentales Agrarias, Instituto Nacional de Innovación Agraria, Jaén 06801, Calamarca, Peru; 5 Dirección de Desarrollo Tecnológico Agrario, Instituto Nacional de Innovación Agraria, Lima, Lima, Peru; 6 Facultad de Ciencias Biológicas, Universidad Nacional Mayor de San Marcos, Lima District, Lima Region, Peru; 7 Escuela Profesional de Ingeniería Agronómica Tropical, Universidad Nacional Intercultural de Quillabamba, Quillabamba, Cusco, Peru; 8 Facultad de Ingeniería y Ciencias Agrarias, National University Toribio Rodriguez de Mendoza of Amazonas, Chachapoyas, Amazonas, 01001, Peru

**Keywords:** Agaricales, cocoa, Marasmiaceae, tropical phytopathogens

## Abstract

The thread blight disease (TBD) of cacao ( *Theobroma cacao*) in the department of Amazonas, Peru was recently reported to be caused by *Marasmius tenuissimus* (sect. Neosessiles). This same species is known to be the main causal agent of TBD in West Africa. However, some morphological characteristics, such as the presence of rhizomorphs, the almost exclusively white color, and pileus sizes less than 5 mm, among others, differ to the description of *M. tenuissimus.* Therefore, we aimed to conduct a taxonomic revision of the cacao-TBD causal agent in Peru, by using thorough micro and macro morphological, phylogenetic, and nuclear and mitochondrial genomic approaches. We showed that the causal agent of TBD of cacao in Amazonas, Peru, belongs to a new species, *Marasmius infestans* sp. nov. This study enriches our knowledge of species in the sect. Neosessiles, and strongly suggests that the *M. tenuissimus* species complex is highly diverse.

## Introduction

*Marasmius* (Marasmiaceae, Agaricales, Agaricomycetes, Basidiomycota) is a hyperdiverse genus of fungi with 1,560 legitimate species names registered in MycoBank, as of Jan. 14 ^th^, 2024. ^1^ It has been traditionally classified into twelve sections, based on morphological characteristics. ^2^ Thus far, molecular re-examination proved that sections *Globulares*, *Marasmius*, *Sicci*, *Leveilleani* and *Neosessiles* belong to *Marasmius* sensu stricto. ^3^^,^^4^ Other sections from the traditional classification, such as *Androsacei*, *Alliacei*, *Epiphylli*, and *Hygrometrici*, have been accommodated outside *Marasmius* s.s., while sections *Fusicystides*, *Inaequales*, and *Scotophysini* have not been yet molecularly studied. ^5^^–^^8^

The great majority of species of *Marasmius* are decomposers of leaves and twigs in natural ecosystems, but some species can be pathogenic in agricultural settings, such as the cacao ( *Theobroma cacao*) agroecosystem. ^9^^–^^11^ Cacao can get infected by several species of *Marasmius*, including *M. crinis-equi* (sect. *Marasmius*) and some species within the section *Neosessiles*, causing thread-blight disease (TBD). ^12^ The sect. *Neosessiles* is a paraphyletic group mainly characterized by the pleurotoid habit of growth and the absence or rudimentarity of the stipe. ^2^^,^^13^ Among the TBD-causing *Neosessiles* species, *M. tenuissimus* seems to be the most frequent in West Africa, ^11^ and in native Awajun and Wampis communities from Northern Peru. ^14^ *Marasmius tenuissimus* is characterized by pilei between 7 and 22 mm in diameter, with rusty brown, light grayish fusco or greyish orange color; and basidiospore dimensions of about 9-10 × 4-6 μm. ^3^^,^^9^ Even though, nuclear rDNA sequence similarities over 99% point to *M. tenuissimus* reference strains, ^3^ the morphological characteristics of specimens from West Africa and Northern Peru do not quite match the original description. ^10^^,^^11^ Pileus sizes less than 5 mm, the white color, and the presence of rhizomorphs, as in the West African and Peruvian specimens, were never reported before for *M. tenuissimus,* ^10^^,^^11^ suggesting they may not be of that species. Moreover, the mitochondrial genome of six strains and the genome of one strain of *M. tenuissimus* from West Africa have been recently published, ^12^^,^^15^ which opens up the door to perform mitochondrial and nuclear genomic comparisons between the *Neosessiles* TBD agents from both continents. Therefore, in this study, we aimed to conduct a taxonomic and phylogenetic revision of the status of the cacao TBD-causal agent in Northern Peru, including nuclear and mitogenomic evidence.

## Methods

### Sampling and morphological analysis

**Figure 1.**
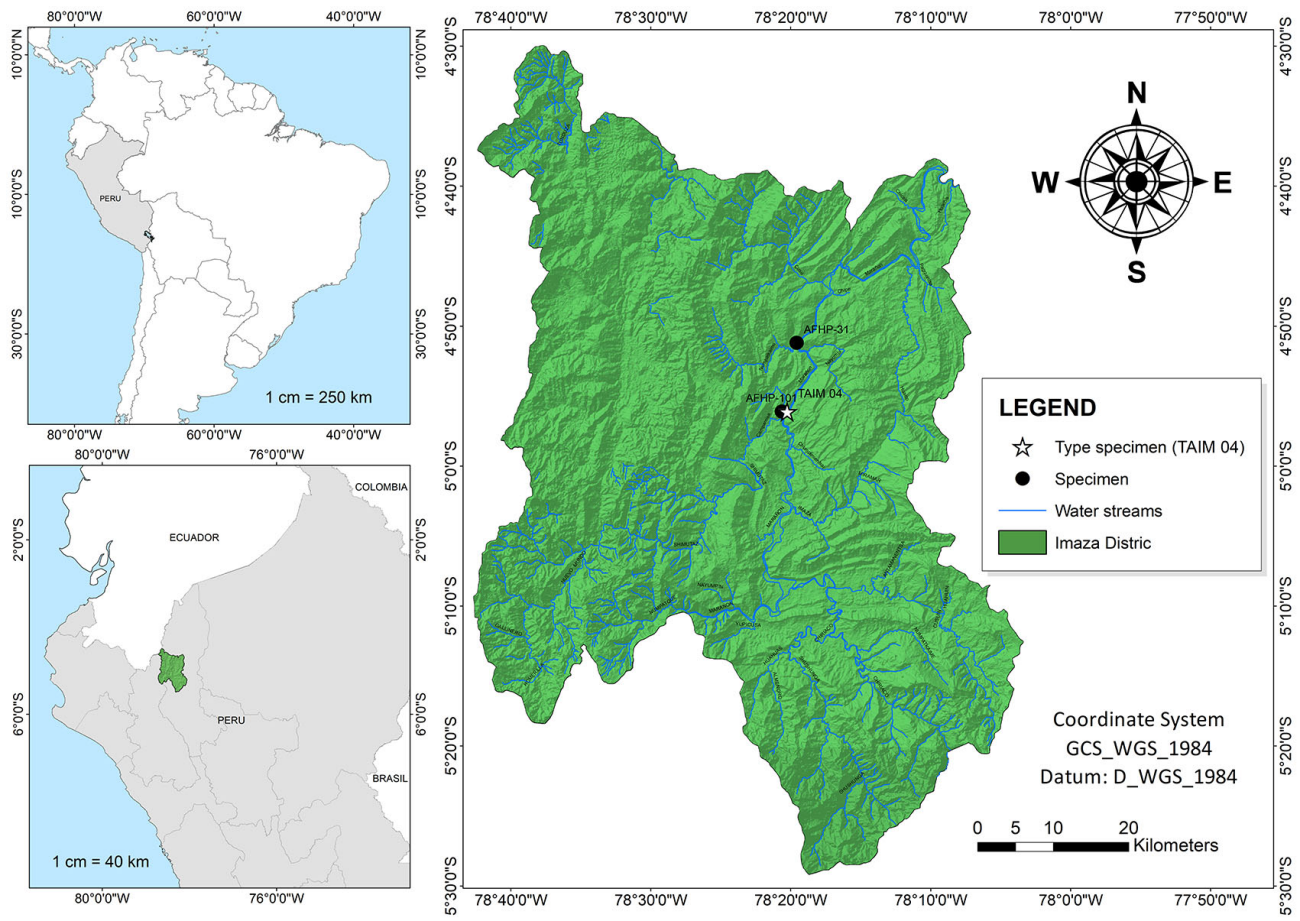
Collection points of specimens in Imaza district, Bagua province, Amazonas department, Peru.

Specimens were obtained during an expedition into the Imaza District (4°47′09.4″S 78°16′51.6″W) in the department of Amazonas, Peru ( Figure 1), during August–September 2022 ( Table 1). Collections were performed under the authorization N° AUT-IFL-2021-052 granted by the Peruvian National Forestry and Wildlife Service Agency - SERFOR. Morphological studies were conducted in the Plant Health Laboratory of the National University Toribio Rodriguez de Mendoza de Amazonas, Peru (UNTRM-A). The macroscopic characterization was made from fresh pilei. We described the color, shape, and size using a stereomicroscope SMZ18 (NIKON, Tokyo, Japan). The microscopic description was made from fresh and dried material. First, a tiny piece of dried material was carefully sectioned and placed onto a microscope slide. We applied 95% ethanol to the sample for 30 seconds, and then a drop of distilled water was added. The slides were dried out and 5% KOH was applied. Additionally, ethanol-washed sections were mounted on Melzer’s reagent as in. ^3^ We placed a cover slip onto the sample and observed it under an OLYMPUS DP74 (Tokyo, Japan) microscope. Dimensions of microstructures were reported with the ranges between the 5 ^th^ and 95 ^th^ percentile, with extreme values in parentheses, as in. ^16^ Additionally, for basidiospores, the arithmetic mean ± standard deviation (x _m_) and the quotient of the length by the width, expressed as range (Q) and arithmetic mean (Q _m_), were calculated. The dimensions of basidia and basidiospores were measured with at least thirty individual structures, i.e., *n* ≥ 30. Finally, pure cultures were obtained from rhizomorph tissues by the hyphal tip technique. The holotype specimen (TAIM04) was deposited in the KUELAP herbarium of UNTRM-A under voucher number KUELAP-2940.

**Table 1.** Taxa used the phylogenetic analysis of this study.

Section	Species	Specimen ID	Origin	Accession number				
				ITS	LSU	*RPB1*	*EF1α*	*COX1*
*Neosessiles*	***Marasmius infestans***	TAIM 04 *	**Peru**	**OR359411**	**OR364495**	**OR420729**	**OR420730**	−
	***Marasmius infestans***	**AFHP-31**	**Peru**	**OM720123**	**OM720135**	**KAK1231915** ^**§**^	**KAK1236186** ^**§**^	**OQ343345** ^**¥**^
	***Marasmius infestans***	**AFHP-101**	**Peru**	**OR359410**	**OR364494**	**–**	**–**	−
	*Marasmius neosessiliformis* nom. prov.	Buyck 97.615	Madagascar	KX149007	−	−	−	−
	*Marasmius tenuissimus*	AKD 304/2015	India	MF189066	−	−	−	−
	*Marasmius tenuissimus*	NW199	Thailand	EU935569	−	−	−	−
	*Marasmius tenuissimus*	NW192	Thailand	EU935568	−	−	−	−
	*Marasmius tenuissimus*	SCAU111	China	MF061773	−	−	−	−
	*Marasmius* sp. 1	RAK 339	Cameroon	MN930548	−	−	−	−
	*Marasmius* sp. 1	GHA07	Ghana	MN794171	−	−	−	UEX92801
	*Marasmius* sp. 1	GHA74	Ghana	MN794173	−	−	−	UEX92859
	*Marasmius* sp. 1	MS2	Ghana	MN794183	MN794074	−	−	UEX92814
	*Marasmius* sp. 1	GHA37	Ghana	MN794147	−	KAJ8088874	KAJ8084062	UEX92895
	*Marasmius* sp. 2	GHA64	Ghana	MN794166	−	−	−	UEX92879
	*Marasmius* sp. 3	GHA63	Ghana	MN794165	MN794071	−	−	UEX92917
	*Marasmius* sp. 4	GHA79	Ghana	MN794177	MN794073	−	−	UEX92836
	*Marasmius* sp. 5	C2/33	Brazil	KM246277	KM246082	−	−	−
	*Marasmius* sp. 5	C2/06	Brazil	KM246261	KM246066	−	−	−
	*Marasmius griseoroseus* var. *diminutus*	JO390 *	Brazil	JX424044	KF742003	−	−	−
	*Marasmius conchiformis* var. *lenipileatus*	JO287 *	Brazil	JX424042	KF742001	−	−	−
	*Marasmius conchiformis* var. *dispar*	JO290 *	Brazil	JX424039	KF742002	−	−	−
	*Marasmius conchiformis*	JO117 *	Brazil	JX424038	KF741998	−	−	−
	*Marasmius griseoroseus*	JO465	Brazil	KJ173479	KJ173480	−	−	−
	*Marasmius conchiformis*	JO45	Brazil	KF741996	KF741997	−	−	−
*Sicci*	*Marasmius nodulocystis*	DED 8278	Madagascar	KX953741	−	−	−	−
	*Marasmius nodulocystis*	DED 8269	Madagascar	KX953740	−	−	−	−
	*Marasmius nodulocystis*	DED 8283	Madagascar	KX953742	−	−	−	−
	*Marasmius linderioides*	JO286 *	Brazil	JX424037	KF742000	−	−	−
	*Marasmius longisetosus*	JO248 *	Brazil	JX424040	KF741999	−	−	−
	*Marasmius siccus*	LE 295985	Russia	KF774132	−	−	−	−
	*Marasmius siccus*	LE 295984	Russia	KF774131	−	−	−	−
	*Marasmius siccus*	VA 08.69	Korea	FJ904992	−	−	−	−
	*Marasmius siccus*	KG 028	Korea	FJ904985	FJ904980	−	−	−
*Marasmius*	*Marasmius* aff. *curreyi*	JES 135	Madagascar	KX149008	−	−	−	−
	*Marasmius curreyi*	Buyck 97.374	Madagascar	KX148980	−	−	−	−
	*Marasmius ruforotula*	TYS438	Malaysia	FJ431271	−	−	−	−
	*Marasmius ruforotula*	TYS369	Malaysia	FJ431269	−	−	−	−
	*Marasmius nigrobrunneus*	TYS281	Thailand	EU935575	−	−	−	−
	*Marasmius nigrobrunneus*	NW223	Thailand	EU935572	−	−	−	−
	*Marasmius nigrobrunneus*	NW162	Thailand	EU935570	−	−	−	−
	*Marasmius gracilichorda*	TYS411	Malaysia	FJ431244	−	−	−	−
	*Marasmius gracilichorda*	TYS396	Malaysia	FJ431242	−	−	−	−
	*Marasmius berambutanus*	TYS398	Malaysia	FJ431227	−	−	−	−
	*Marasmius berambutanus*	TYS337	Malaysia	FJ431225	−	−	−	−
	*Marasmius graminum*	NN005953	Denmark	JN943595	JN941141	−	−	−
	*Marasmius graminum*	FO 46723	Germany	−	AF291345	−	−	−
	*Marasmius crinis-equi*	GHA76	Ghana	MN794174	MN794072	−	−	UEX92959
*Leveilleani*	*Marasmius leveilleanus*	NW268	Thailand	EU935567	−	−	−	−
	*Marasmius leveilleanus*	NW248	Thailand	EU935566	−	−	−	−
	*Marasmius* aff. *leveilleanus*	RAK 392	Cameroon	MN930527	−	−	−	−

^*^ Taxa marked with an asterisk are type specimens of the corresponding species.

^**§**^ These accession numbers correspond to the translated coding sequences of *RPB1* and *EF1α*. The DNA sequence of *RPB1* sequence is located from position 29369 to 32406 of JANHQD010001077.1; *EF1α* sequence from positions 10248 to 12091 of JANHQD010000042.1.

^**¥**^ This accession number correspond to the accession number of the mitochondrial genome. COX1 sequence is located from position 43,391 to 44,992.

### Phylogenetic analysis

DNA extractions were performed from pure cultures using the Wizard® genomic DNA purification kit (Catalogue number A1120; Promega, Wisconsin, USA). DNA was quantified with the BioSpectrometer® Basic (Eppendorf, New Jersey, USA), and diluted to 0.5 ng/μl for PCR reactions. The internal transcribed spacer region (ITS1-5.8S-ITS2, or simply ITS), the large subunit (LSU) rDNA gene, and partial fragments of the genes Elongation Factor 1-α ( *EF1α*) and the largest subunit of RNA polymerase II gene ( *RPB1*) were amplified following. ^17^ The amplified PCR products were Sanger sequenced at MACROGEN (Seoul, South Korea). LSU, *EF1α,* and *RPB1* sequences from the West African TBD-causing specimen GHA37 were retrieved from the recently published genome. ^15^ In addition, we included the mitochondrial cytochrome oxidase I gene ( *COX1*) from isolate INDES-AFHP31, which was retrieved from the mitogenome generated in this study, and from other phylogenetic-related species. ^12^ The introns of *COX1* sequences were removed as in. ^18^ Most other sequences used in phylogenetic analyses were obtained from other relevant literature. ^3^^,^^4^^,^^11^^,^^13^^,^^14^^,^^19^^–^^25^ We also included unpublished sequences available in NCBI from *M. tenuissimus*-phylogenetically related isolates C2/06 and C2/33 (accession numbers KM246261 and KM246277 for ITS, and KM246066 and KM246082 for LSU, respectively).

Generated sequences were edited and assembled with Sequencher v.5.4 (Gene Codes, USA). Once all sequences were gathered, they were aligned with MUSCLE ^26^ implemented in MEGA-X, ^27^ and concatenated with SeaView 4.7. ^28^ We used jModelTest v2 ^29^ to identify the most appropriate nucleotide evolution models under the Akaike information criterion. Phylogenetic analysis was performed with IQ-TREE v2 ^30^ which implements the Maximum Likelihood algorithm, ^31^ in the CIPRES Science Gateway v3.3 portal. ^32^ The phylogenetic trees were visualized and edited with FigTree. ^33^

### Genomic analysis

**DNA extraction, sequencing, and assembly**

Total DNA extraction was performed using the Wizard Purification Kit (Promega Corp., Madison, Wisconsin) following the manufacturer’s instructions. For nuclear and mitogenome sequencing, we used the strain INDES-AFHP31 from Northern Peru, previously reported. ^14^ A paired-end sequencing library was constructed using the TruSeq Nano DNA Kit, according to the manufacturer’s instructions. The library was sequenced on an Illumina NovaSeq 6000 platform in paired-end, 2 × 150 format.

The raw reads were checked by FastQC v.0.11.9. Also, quality trimming (Phred Q > 25) and remotion of adapters were conducted with Trimmomatic v0.36 ^34^ and TrimGalore software, ^35^ respectively. Jellyfish v.2. ^36^ was used for k-mer counting, and Genome Scope v1.0.0 ^37^ for assessing genome size, repeat content, and heterozygosity rate. This analysis involved utilizing the output of Jellyfish and the count of 17-mers for k-mer analysis. Additionally, k-depth estimation was performed to identify a predominant single-peak pattern in the frequency distribution analysis of k-mers.

De novo assembly was performed with two assembly algorithms: SOAPdenovo2 v.2.04, ^38^ and Masurca v.4.0.6. ^39^ Next, we used QUAST v.5.2.0 ^40^ to evaluate the statistics of assemblies. The assembly validation process employed two distinct methods. First, the filtered paired-end Illumina reads were realigned to identify any errors in the assembly. This was accomplished using Bowtie2 v.2.4.2 ^41^ and SamTools v.1.7 ^42^ software. Second, the completeness of the assembly was evaluated using the Agaricales-specific profile of the BUSCO strategy. ^43^ To identify vector contamination, we employed VecScreen JCVI ( https://github.com/tanghaibao/jcvi), which utilizes the Univecdatabase ( https://ftp.ncbi.nlm.nih.gov/pub/UniVec/). We also performed a BLAST v.2.2.26 ^44^ analysis, mapping the scaffolds against the nt/nr NCBI database (found at https://www.ncbi.nlm.nih.gov/). Following this, any contaminated scaffolds and vectors were eliminated before submitting the remaining data to the NCBI database. This assembly has been deposited at DDBJ/ENA/GenBank under the accession: JANHQD01.

**Nuclear genome annotation**

Genome annotation was performed using Funannotate v1.8.12. ^45^ Repetitive elements were soft-masked by TANTAN. ^46^ *Ab initio* gene prediction was carried out using AUGUSTUS v.3.3.3, ^47^ GlimmerHMM v.3.0.4, ^48^ and SNAP ^49^ trained with alignments of the BUSCO agaricales_odb10 dataset, ^46^ and with GeneMark-ES v.4.69 ^50^ self-trained on the repeat-masked genome sequence. Protein evidence from the UniProt/SwissProt database ^51^ was mapped to the genome using DIAMOND v.2.0.15 ^52^ and Exonerate v.2.4.0. ^53^ Finally, EVidenceModeler v.1.1.1 ^54^ was used to generate consensus gene models based on all the above ab initio and evidence-based gene models. The tRNA genes were identified with tRNAscan-SE v.2.0.9. ^55^ Functional annotations were assigned by similarity to UniProtKB (2021_02), ^51^ InterPro v89.0, ^56^ Pfam v.34.0, ^57^ EggNOG v.5.0, ^58^ BUSCO (agaricales_odb10 dataset), ^43^ dbCAN v.10.0, ^59^ and MEROPS v.12.0. ^60^ Phobius ^61^ and SignalP v.5b ^62^ were used to predict transmembrane topology and signal peptides, respectively. DeepLoc 2.0 ^63^ was used to determine protein localization. Effectors were predicted by EffectorP 3.0 ^64^ based on the subset of extracellular proteins. AntiSMASH v.6.0 ^65^ was used to predict secondary metabolite gene clusters (SMGCs) and secondary metabolite Clusters of Orthologous Groups (smCOGs).

**Mitochondrial genome annotation**

The mitochondrial genome was confirmed using the default Geneious Prime 2023.1 setup (Biomatters Ltd., Auckland, New Zealand) and GetOrganelle v1.7.6.1. ^66^ Genes were annotated with MITOS, ^67^ MFannot ^68^ and manually confirmed with ORFfinder available in NCBI, and tRNAscan-SE 1.21, ^69^ finally adjusted in Geneious. A physical map of the mitogenome was created with OGDRAW v 1.2. ^70^ Our results were compared to the ones reported for another published *M.* aff. *tenuissimus* mitogenomes. ^12^

## Results

### Sampling and morphological analysis

**Figure 2.**
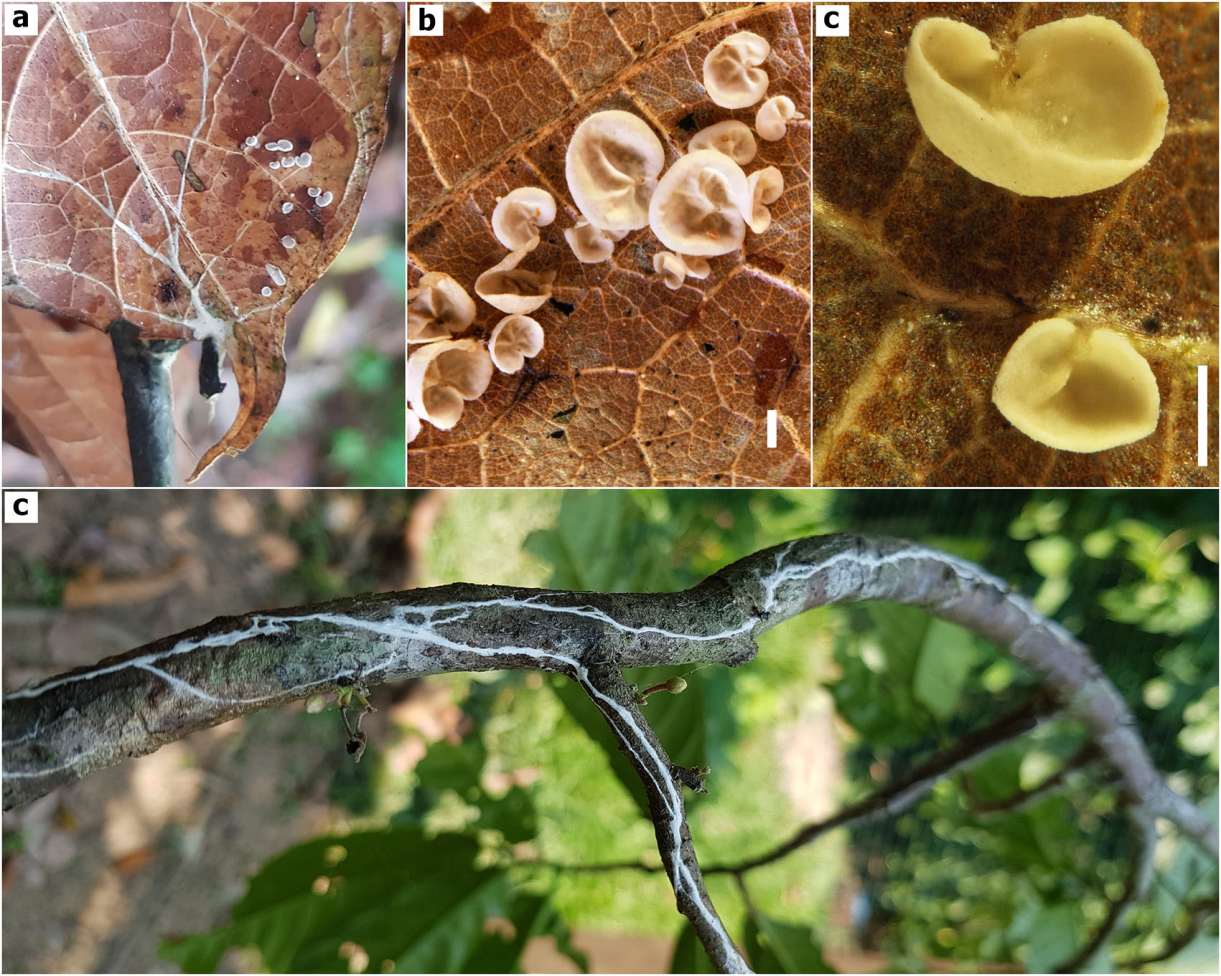
Macroscopic features *Marasmius infestans* sp. nov. (TAIM 04, holotype). a) Small basidiocarps growing on decomposing cacao leaves, b) Mature basidiomata, c) Young basidiomata, d) White-rhizomorphs colonizing cacao stem. Photographed by A. F, Huaman-Pilco. Scale bar in b) and c) = 1 mm.

**Figure 3.**
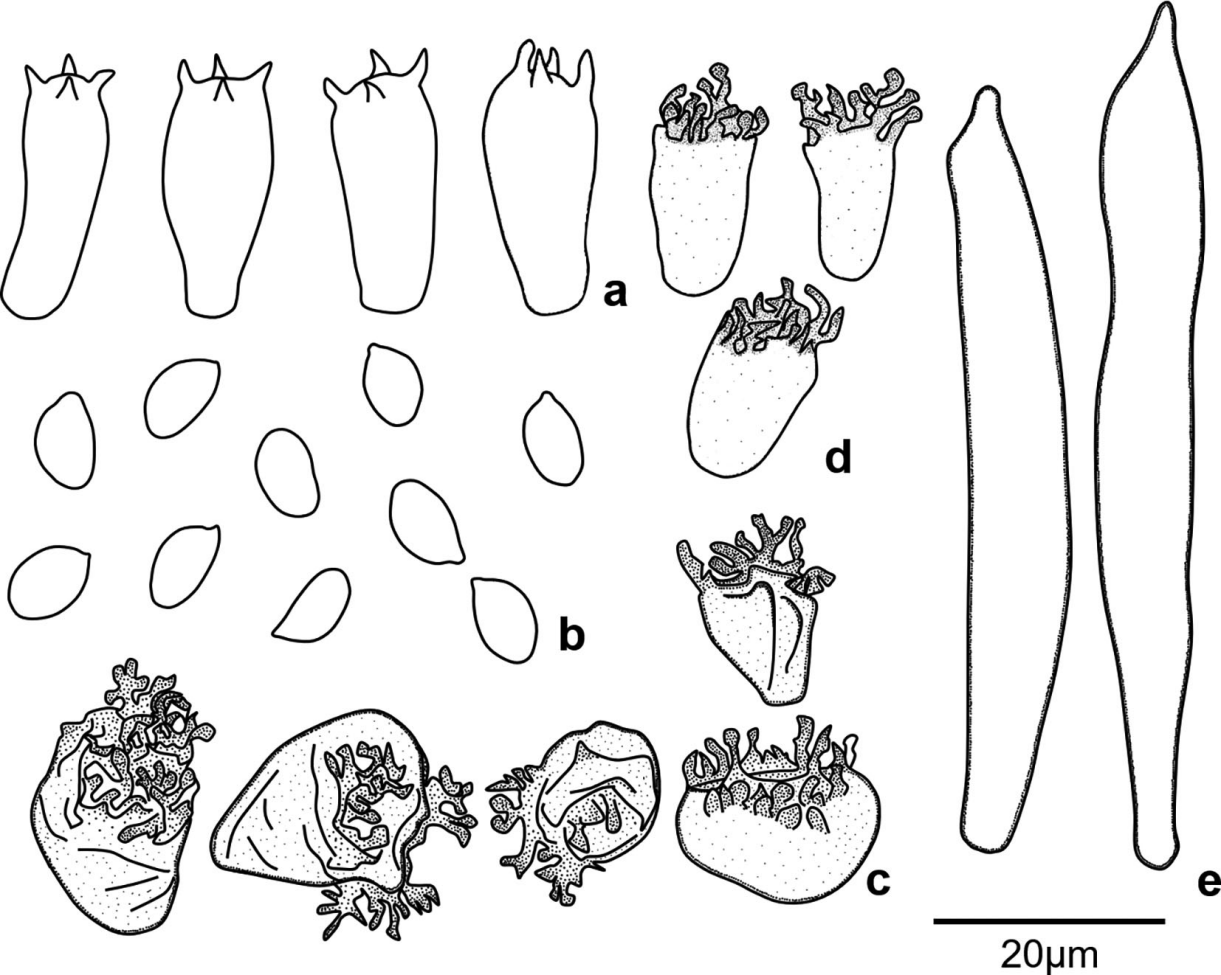
Microscopic features of *Marasmius infestans* sp. nov. (TAIM 04, holotype), a) Basidia, b) Basidiospores, c) *Siccus*-type broom cells of Pileipellis, d) Cheilocystidia of *Siccus*-type broom cells, e) Caulocystidia. Scale bar = 20 μm. Drawings by T. A. Ramos-Carrasco.

**Figure 4.**
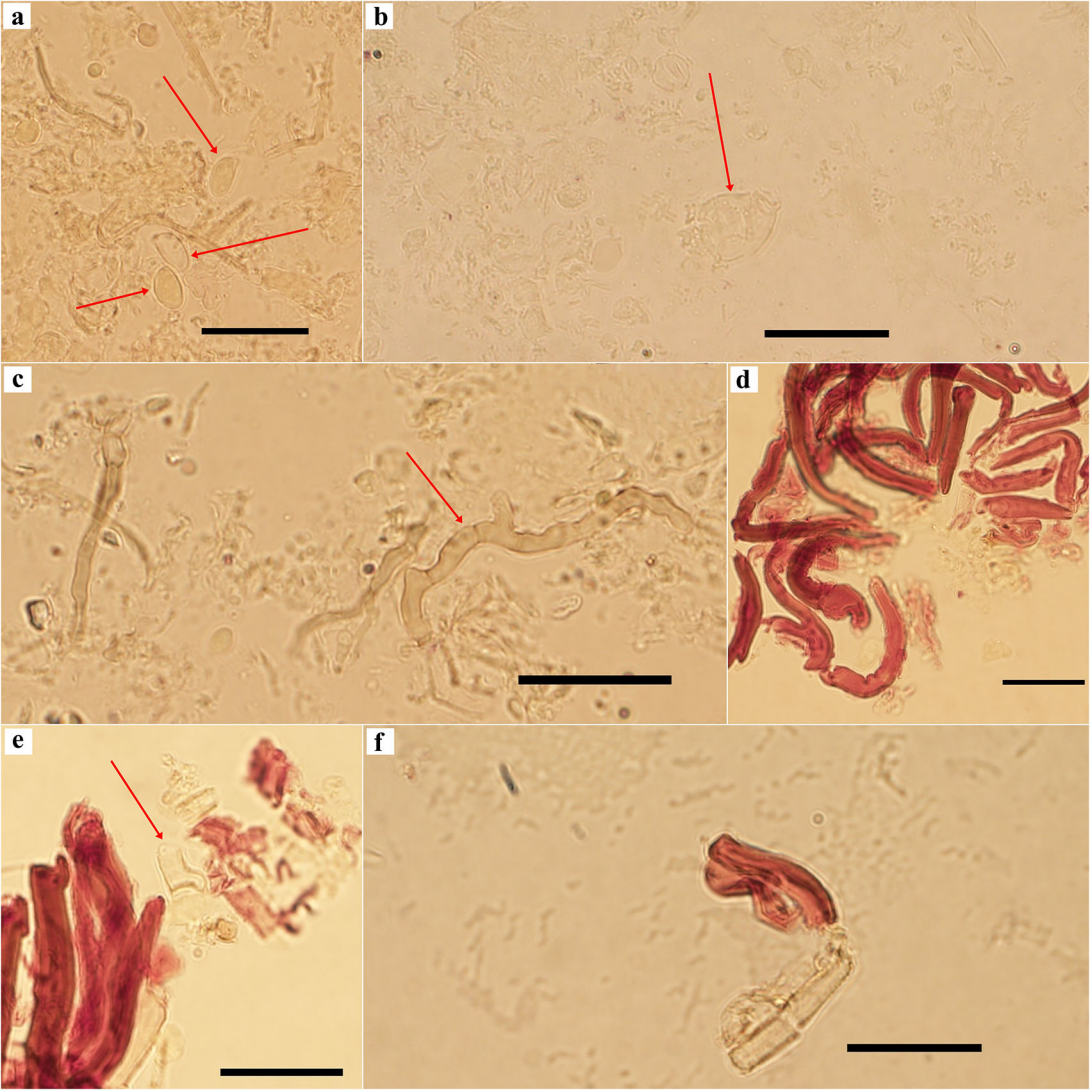
Melzer’s reagent examination of *Marasmius infestans* sp. nov. (TAIM 04, holotype), a) Inamyloid basidiospores, b) Inamyloid pileipellis broom cell, c) Inamyloid pileus trama hyphae, d) Strongly dextrinoid caulocystidia, e-f) Inamyloid stipitipellis hyphae and caulocystidia.

We collected new cacao tissues infected by TBD-bearing fruiting bodies of the pathogen in Imaza province, department of Amazonas, in Northern Peru. Besides AFHP-31 from a previous study, ^14^ and for which no basidiocarps and only pure agar culture were preserved, two additional specimens were included in this study: TAIM-04 and AFHP-101. The presence of white rhizomorphs, and white pilei no larger than 5 mm in diameter, smooth and non-intervenose are macro morphology hallmarks of the TBD- causing specimens in Amazonas, Peru ( Figure 2). In terms of micromorphological features, these specimens produce ellipsoid and smooth basidiospores with dimensions of (6.2-)7.0-8.6(-8.8) × (3.7-)3.8-5.0(-5.2) μm; they also produce *Siccus*-type cheilocystidia and pileipellis broom cells; cheilocystidia are cylindrical slightly narrower at the base, and pileipellis broom cells are ovoid to globose shape ( Figure 3). The examination of tissues in Melzer’ reagent revealed that most micro structures are inamyloid, except for caulocystidia which reacted in a strongly dextrinoid manner ( Figure 4). All these morphological features provided the first point of evidence that the cacao TBD-causal agent in Peru is a new species.

### Phylogenetic analysis

**Figure 5.**
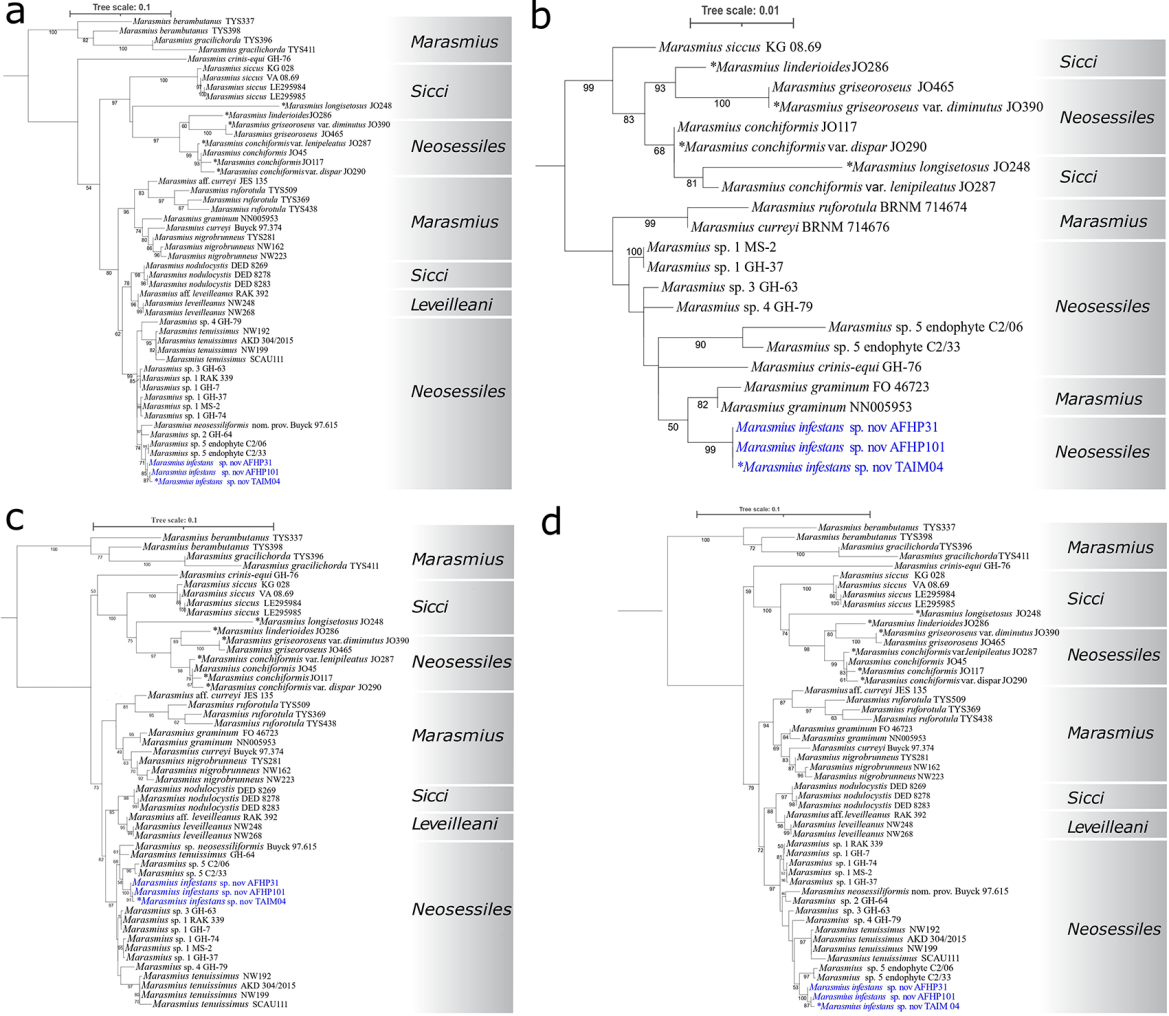
Phylogenetic analyses of *Marasmius* taxa in the *Neosessiles* and other closely related sections conducted in this study. a) Phylogenetic analysis with the internal transcribed spacer (ITS1-5.8S-ITS2, ITS) rDNA region using the Hasegawa-Kishino-Yano nucleotide substitution model with a discrete Gamma model for the rate of heterogeneity (HKY + G). b) Phylogenetic analysis using the large subunit (LSU) rDNA region using the transition model 3 with equal frequency allowing for proportion of invariable sites (TIM3+I). c) Phylogenetic analysis with the ITS-LSU concatenated dataset using the transversion model with unequal base frequency with invariable site plus discrete Gamma model for the rate of heterogeneity (TVM + I +G). d) Phylogenetic analysis with the multi-locus dataset composed by ITS, LSU, and partial sequences of the Translation Elongation Factor 1-α ( *EF1α*), the largest subunit of RNA polymerase II gene ( *RPB1*), and the mitochondrial cytochrome oxidase I ( *COX1*) genes, using the transversion model with unequal base frequency, with a discrete Gamma model for the rate of heterogeneity (TVM + G). All phylogenetic trees were midpoint-rooted and built under the maximum likelihood framework. Models of evolution were determined by jModelTest2. ^29^ Values above branches = Maximum likelihood bootstrap values. Taxa in blue are the specimens causing thread-blight disease of cacao in Amazonas, Peru, and conform a new species clade, *Marasmius infestans* sp. nov. Type specimens are marked with an asterisk.

We used fifty taxa (type and other reference specimens) of *Maramius* spp. in the *Neosessiles* and other closely related sections. As expected for paraphyletic groups, the phylogenetic analysis grouped sect. *Neosessiles* taxa in two different parts of the trees built with the ITS, LSU, ITS-LSU concatenated dataset, and six-gene-multilocus concatenated dataset ( Figure 5). ^13^^,^^24^ The Peruvian specimens were grouped together in a highly supported clades in all phylogenetic analyses (85–100% bootstrap support; Figure 5). The closest related clade was composed of the unpublished *Marasmius* isolates (C2/33, C2/06), putatively soybean endophytes according to their NCBI passport information. These two clades were phylogenetically distinct to the *M. tenuissimus* sensu stricto clade conformed by the reference strains NW192, and NW199, and other reference specimens ( Figure 5). Therefore, these results, combined with the unique morphological characteristics of the Peruvian specimens, support they belong to a new species within the sect. *Neosessiles*, herein after called *Marasmius infestans* sp. nov.

West African specimens were phylogenetically distinct to both, *M. tenuissimus* sensu stricto, and the Peruvian-specimen clade ( Figure 5). They were grouped in at least four phylogenetic clades. The informally described *M. neosessiliformis* nom. prov. was grouped together with isolate GHA64.

### Nuclear and mitochondrial genomic analyses

The nuclear genome assembly of *Marasmius infestans* AFH-31 reveals a size of 84.7 Mb, organized in 3,213 contigs (≥1,000 bp) with a GC content of 49.32% and a N50 value of 42,194 kb. We predicted 21,762 protein-coding genes and 656 tRNA genes in the nuclear genome of *M.* *infestans* strain AFHP31. Functional annotation resulted in the identification of 15,414 Pfam domains; 29,349 InterPro protein families; 31,346 Clusters of Orthologous Groups of proteins (COGs) and EggNog proteins; 606 proteases/protease inhibitors, and 679 carbohydrate-active enzymes (CAZymes). Moreover, we predicted 1,823 signal peptides and 4,010 transmembrane regions. About 8.9% of the proteome (2,152) are extracellular proteins, of which 25.6% (550) and 16.5% (356) were predicted to be apoplastic and cytoplasmic effectors, respectively. The genome contained 11 SMGCs, 18 biosynthetic enzymes, and 27 smCOGs. The draft nuclear genome of *M. infestans* AFHP-31 is larger in size than isolate GHA-37 genome size and, despite having fewer proteins, has more effectors ( Table 2).

**Table 2.** Comparison of nuclear genomes with the phylogenetic close specimen from West Africa, isolate GHA37.

Genomic characters	*M. infestans* (INDES-AFHP31)	*Marasmius* sp. 1 (GHA37) ^15^
Genome Size	84,695,575	71,059,514
Protein-coding genes	21,762	24,991
Number of effectors prediction	906	675
Number of genes with peptide signal	1,823	1,900

Data for isolate GHA37 obtained from. ^15^

**Figure 6.**
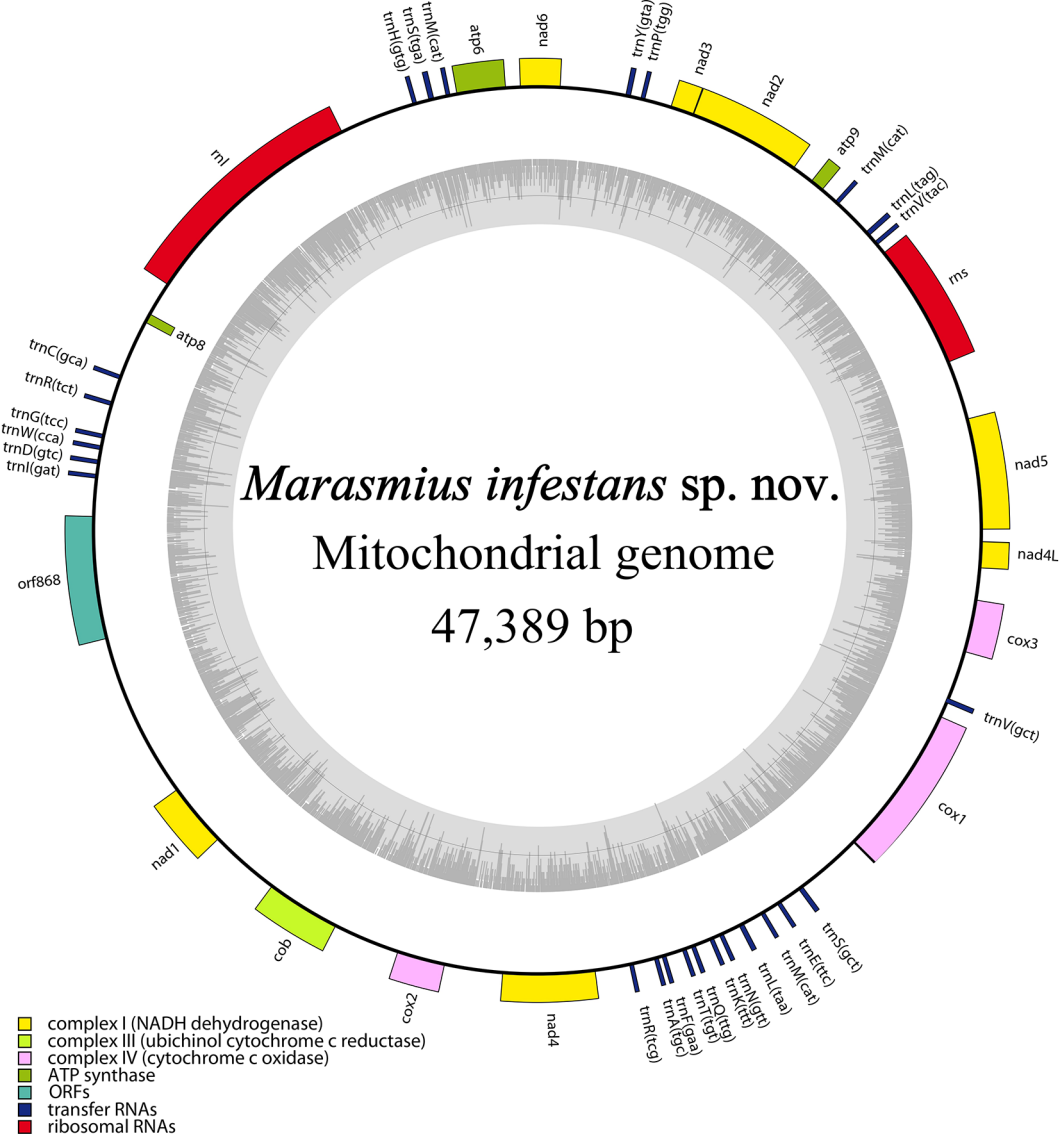
Circular map of *Marasmius infestans* sp. nov. mitochondrial genome. The genes inside and outside the circle are transcribed in clockwise and counterclockwise directions, respectively. Genes belonging to different functional groups are shown in different colors. The dark gray area in the inner circle denotes GC content.

The mitochondrial genome of *Marasmius infestans* is circular, 47,389 bp long, and contains 42 genes. It is A + T rich (72.83%) and includes 25 tRNA (trnR, trnL and trnS occur in duplicate, while trnM in triplicate), 14 ribosomal proteins, two rRNA (rnl, rns), and one orf (orf868) ( Figure 6). A comparison with the mitochondrial genome of TBD-causal agents from West Africa reveals *M. infestans* differs to them in size, and gene number, and has lost all their introns in *COX1* ( Table 3).

**Table 3.** Comparison our *M. infestans* with mitochondrial genomes available in GenBank. ^12^

Species	Isolate/Specimen	Length (pb)	% GC	No of tRNAs	No. Protein-coding genes	No. of introns in COX1
*Marasmius infestans*	AFHP31	47,389	27.17	25	42	0
*Marasmius* sp. 1	GHA07	44,621	27.04	25	17	1
*Marasmius* sp. 1	GHA74	44,859	27.01	25	17	1
*Marasmius* sp. 1	MS2	44,524	27.02	25	18	1
*Marasmius* sp. 1	GHA37	44,399	26.97	25	18	1
*Marasmius* sp. 2	GHA64	43,121	26.66	25	19	1
*Marasmius* sp. 3	GHA63	51,210	27.21	25	21	3
*Marasmius* sp. 4	GHA79	48,952	26.82	26	21	3

Data for *Marasmius* sp. 1–4 taken from. ^12^

**Taxonomy:**

***Marasmius infestans*** Huamán, Ramos C. & Díaz Val., **sp. nov.** IF 901138 ( Figures 1-2).

***Etymology:*** ‘ *infestans*’, in reference to the capacity to infect branches and leaves of cacao trees.

**Diagnosis:** Similar to *Marasmius tenuissimus* and *M. neosessilis* but *M. infestans* has white to white-cream pilei at young and mature stages, lamella non-intervenose, and presence of ovoid to globose pileipellis broom cells. It is also phenetically similar to *M. griseoroseus* but *M. infestans* lacks *Rotalis-* and *Amyloflagellula-*type pileipellis broom cells and pileus-trama is inamyloid.

***Type:*** PERU: Amazonas department, Bagua province, Imaza district, Pumpu native community; -4.785944, -78.281000; leg. Tito Ramos-Carrasco; Sept. 2022. Holotype specimen TAIM-04 (voucher KUELAP-2940).

***Other examined specimens:*** AFHP-101 and dried culture of AFHP-31 (voucher KUELAP-2251).

***Pileus:*** (0.7-)1.1-4.0(-4.9) mm diam., convex, smooth, glabrous, dull to shiny, white to white-cream ( *n* = 84). *Context* white, thin. *Lamellae* adnate, distant (1-5) with 0 series of lamellulae, narrow, smooth, non-intervenose, white, non-marginate. ***Stipe:*** absent or extremely rudimentary, eccentric, cylindrical, insititious not arising from rhizomorphs; white rhizomorphs frequently present. *Odor* not distinctive.

***Basidiospores:*** (6.2-)7.0-8.6(-8.8) × (3.7-)3.8-5.0(-5.2) μm [x _m_ = 7.8± 0.5 × 4.6 ± 0.4 μm, Q = 1.4-2.0, Q _m_ = 1.7, *n* = 45], hyaline, inamyloid, ellipsoid, smooth, thin-walled ( *n* = 44). ***Basidia:*** length (17.3-)17.7-21.5(-22.2) μm, thicker part (7.0-)7.3-10.1(-10.2) μm, thinner part (2.6-)3.1-6.1(-6.3) μm, hyaline, inamyloid, cylindrical to clavate ( *n* = 30). ***Cheilocystidia:*** *Siccus-*type broom cells, hyaline, inamyloid; main body 17.8-20.6 × 7.3-9.9, cylindrical, slightly narrower at the base, thin-walled; apical setulae length 2.3-3.8(-4.1) μm, obtuse, thin-walled. ***Caulocystidia:*** setoid, hyaline, strongly dextrinoid, thin- to thick-walled, main body length (41.7-)46.5-85.1(-87.5) μm, thicker part (7.4-)7.6-10.5(-10.9) μm, thinner part 3.0-5.0(-5.1) μm ( *n* = 5). ***Pileipellis:*** hymeniderm, mottled, composed of *Siccus*-type broom cells; main body (9.6-)9.8-12.1(-12.2) × (8.5-)8.9-16.8(-17.9) μm, ovoid to globose, light-brown to hyaline, thin- to thick-walled ( *n* = 7). ***Pileus trama:*** interwoven, inamyloid. ***Lamellar trama:*** hyphae (2.4-)2.6-3.9(-4.0) μm diam., hyaline, inamyloid, non-gelatinous. ***Stipitipellis:*** hyphae diam 3.4-4.6 μm., hyaline, inamyloid, non-gelatinous. ***Clamp connections:*** present in all tissues.

***Habit and habitat:*** Rhizomorphic colonizing leaf and twigs of cacao, which subsequently causes thread blight disease. Rhizoids are white; fruiting bodies develop on dead tissue. The fungus occurs on poorly managed cacao farms with excessive shade and high relative humidity, typical characteristics of the tropical rainforest.

***Geographic distribution*** Imaza province, Amazonas department, Peru.

***Notes:** Marasmius infestans* differs from *M. tenuissimus* in the color and size of the pilei, and lamellae appearance. *Marasmius infestans* pilei are white to white-cream, while *M. tenuissimus* is between light grayish fuscous to rusty brown when fresh, ^9^ or greyish or pale orange to golden brown, light brown or orangish white. ^3^^,^^4^^,^^9^ Pilei in *M. infestans* can reach up to 5 mm broad, while pilei in *M. tenuissimus* can have diameters typically of 7–40 mm. ^3^^,^^4^ Lamellae in *M. infestans* lacks reticulation while in *M. tenuissimus* lamellae is heavily reticulate. Additionally, West African species within the *M. tenuissimus* species complex can produce pure-white to brown-colored rhizomorphs, ^11^ while *M. infestans* produces pure-white rhizomorphs. At the genomic level, *M. infestans* is 13,636,061 pb larger than *M.* aff. *tenuissimus* GHA37. *Marasmius infestans* also has 3,229 fewer genes, and 231 more effectors ( Table 2). Moreover, *M. infestans* differs from *M. neosessilis* and its varieties ( *M. neosessilis* var. *neosessilis* and *M. neosessilis* var. *montepiensis*) in that these taxa can have various pilei colors such as apricot, salmon-flesh, and gray. ^9^^,^^71^ Also, *M. neosessilis* and the other phenetically similar species *M. griseoroseus* have pseudoamyloid (dextrinoid) pileus-trama, while pileus-trama of *M. infestans* is inamyloid.

## Discussion

The causal agent of cacao TBD had been previously analyzed based on nuclear rDNA comparison, macro morphology of the fruiting body and rhizomorphic structures, and mycelial culture. ^14^ However, other important characteristics, such as the micro morphological features of lamella and pileus, were not considered in reports from both, West Africa and Peru. ^11^^,^^14^ In this study, we present morphological, phylogenetic, and genomic evidence that the species of *Marasmius* causing TBD in the Amazonian areas of Northern Peru is a new species in the sect. *Neosessiles*, namely *M. infestans.*

In this study we revealed morphological differences with *M. tenuissimus* s.s. in color and size of pilei, lamellae appearance, and growth habit. *Marasmius infestans* differs from *M. tenuissimus* mainly because of their smaller basidiocarps. With respect to color, *M. infestans* can be easily distinguished by its white to white-cream pilei. Also, *M. tenuissimus* has never been reported to colonize the leaves and stems of plants with abundant white rhizomorphs as *M. infestans.* ^9^^,^^72^ Additionally, other relates species with pleurotoid-habit of growth such as *M. neosessilis, M. griseoroseus, M. conchiformis*, and *M. jasingensis* also differ in the color of pilei, which can go from gray and pale orange to brown and reddish-brown at maturity. ^9^^,^^13^^,^^73^ The most macro morphological similar species to *M. infestans* is *M. griseoroseus:* pilei are most frequently no more than 5 mm broad, and lamellae is adnate and distant, just as *M. infestans. Marasmius griseoroseus* can also have white to white-cream pilei. ^9^^,^^13^ However, pilei in *M. griseoroseus* can be pale orange as well. Also, several micro morphological characteristics, such as the presence of both *Rotalis-* and *Amyloflagellula*-type pileipellis broom cells, and some degree of dextrinoidity in pileus trama are marked differences with *M. infestans.* ^9^^,^^13^ Moreover, the informally described species *M. neosessiliformis* nom. prov. is one of the few very closely related taxa for which there is ITS sequence data available in GenBank. ^19^ The provisional description of this species pointed to several shared characteristics with *M. infestans*, such as the size and the non-intervenose nature of pilei, and the presence of *Siccus*-type broom cells in the pileipellis. However, it differs in that *M. neosessiliformis* nom. prov. has much larger basidiospores (10–11 × 5–6 μm) than *M. infestans*. ^19^

In West Africa, four different species were reported to cause TBD of cacao: *M. crinis-equi*, *M.* aff. *tenuissimus*, *M. scandens,* and *Paramarasmius palmivorus* (reported as *Marasmius palmivorus*). ^11^ Besides the molecular differences, these TBD causal agents presented five rhizomorph morphotypes. ^11^ The morphotype A of rhizomorphs is characterized by abundant thin, black, “horsehair”-type rhizomorphs, and was only found on *M. crinis-equi.* The morphotype B is characterized by its brown coloration; and the type C, by its intense white color. Both morphotypes B and C, were found on *M.* aff. *tennuisimus.* The morphotype D is characterized by a faint cream or dull white rhizomorph, with the presence of smooth or cream-pruinose pilei, with a diameter up to 8 mm; and the morphotype E is characterized by its aggregation of shiny or silky white hyphae and white or pale yellow basidiocarps, with smooth and convex pilei, with diameter from 10 to 50 mm, observed on *P. palmivorus.* ^11^ If we followed this classification in our study, we find *M. infestans* has rhizomorphs of morphotype C. Unfortunately, in the West African study, no fruiting bodies from this type of rhizomorphs were reported, so we cannot make a macro morphological comparison.

In the *Marasmius* genus, ITS has been the main locus for phylogenetic studies of species in sect. *Neosessiles.* ^3^ In this study, we found *M. infestans* is closely related species to *M. tennuisimus* s. s., both species forming individual clades with high bootstrap support (>85%) in all phylogenetic analyses conducted. We also found that the *M. tenuissimus* is a species complex that has at least five other species that will need proper description, including the previously reported yet not formally described *M. neosessiliformis* nom. prov., ^19^ provided the discovery of corresponding basidiocarps. One of these species ( *Marasmius* sp. 5) corresponds to the endophytic strains C2/33 and C2/06 from soybean in Brazil forming a distinct phylogenetic clade, with 97% bootstrap support and closely related to *M. infestans* and *M. tennuisimus.* Additionally, TBD-causal strains from West Africa form separate and well-supported clades. Isolate GHA64 groups together with the informally described species *M. neosessiliformis* nom. prov. specimen Buyck 97.615, suggesting it may be another member of this species requiring formal description.

On top of morphological and phylogenetic evidence that *M. infestans* distinguishes from other species within the sect. *Neosessiles*, in this study we present its nuclear and mitochondrial genome sequences *.* The nuclear genome is about 13 Mb longer than *M.* aff. *tennuisimus* GHA37. *Marasmius infestans* also has 231 more effectors than *M.* aff. *tennuisimus* GHA37. Pathogenic effectors are secreted by pathogenic fungi during infection and play an important role in silencing plant defenses response, ^64^^,^^74^^,^^75^ which may help *M. infestans* during cacao infection *.* Moreover, mitochondrial genomes are known for different sizes and rearrangements despite their similar gene function. ^12^^,^^76^ *Marasmius infestans* mitogenome has 47,389 bp and has undergone the loss of introns in the COX1 gene, as opposed to *M.* cf. *tenuissimus* causing TBD in Africa ( Table 3). The gain and loss of introns are common in fungal mitogenomes and are related to their evolution . ^76^ Moreover, we found that *M.* cf. *tennuisimus* specimens GHA74, GHA37, MS2, and GHA07, which group together in the phylogenetic analyses with the six-gene-multilocus dataset, have very similar mitochondrial genome sizes, ranging from 44,399 to 44,859 bp, supporting they all belong to another species ( *Marasmius* sp. 1; Figure 5). Additionally, specimens GHA63, and GHA79, which also formed individual specific lineages, have different mitochondrial genome sizes (51,210 and 48,952 bp, respectively). ^11^ Therefore, they also represent other *Neosessiles* species in need of formal description ( *Marasmius* sp. 3 and *Marasmius* sp. 4, respectively).

## Data Availability

BioProject: The Genome Shotgun project of specimen AFHP-31. Accession number PRJNA860982; https://identifiers.org/NCBI/bioproject:PRJNA860982 The NCBI accession number of the version of the assembled genome of specimen AFHP-31 described in this paper is JANHQD010000000. Nucleotide: The mitochondrial genome assembly. Accession number OQ343345; https://www.ncbi.nlm.nih.gov/nuccore/OQ343345.1/ SRA: The raw reads for the nuclear and mitochondrial genome under accession number SRR20354643. https://identifiers.org/insdc.sra:SRR20354643 Sequences obtained through the Sanger method used are deposited at NCBI through accession numbers OR359411, OR364495, OR420729 and OR420730 for specimen TAIM-04; OM720123 and OM720135 for specimen AFHP-31; and OR359410 and OR364494 for specimen AFHP-101.
