# Supplementary material for: Morphological, phylogenetic, and genomic evidence reveals the causal agent of thread blight disease of cacao in Peru is a new species of Marasmius in the section Neosessiles, Marasmius infestans sp. nov
Source: F1000Res. 2024 Jan 29;12:1327. Originally published 2023 Oct 12. [Version 2] doi: 10.12688/f1000research.140405.2 (PMC11053350; doi:10.12688/f1000research.140405.2)
Supplement: Peer review history

# Reviewer report: Reviewer response for version 2

**DOI:** 10.5256/f1000research.161562.r241843

**Published:** 2024-04-26

**Authors:** Nopparat Wannathes

**Affiliations:** 1 Microbiology Program, Faculty of Science and Technology, Pibulsongkram Rajabhat University, Phitsanulok, Phitsanulok, Thailand

No further comments.

Is the work clearly and accurately presented and does it cite the current literature?

Partly

If applicable, is the statistical analysis and its interpretation appropriate?

Partly

Are all the source data underlying the results available to ensure full reproducibility?

No

Is the study design appropriate and is the work technically sound?

Partly

Are the conclusions drawn adequately supported by the results?

Partly

Are sufficient details of methods and analysis provided to allow replication by others?

Yes

Reviewer Expertise:

Taxonomy and systematics on agaric

I confirm that I have read this submission and believe that I have an appropriate level of expertise to confirm that it is of an acceptable scientific standard.

# Reviewer report: Reviewer response for version 2

**DOI:** 10.5256/f1000research.161562.r241844

**Published:** 2024-04-26

**Authors:** Atik Retnowati

**Affiliations:** 1 Herbarium Bogoriense, Cibinong, Indonesia

The revised article has met the reviewer's comments.

Is the work clearly and accurately presented and does it cite the current literature?

Yes

If applicable, is the statistical analysis and its interpretation appropriate?

Yes

Are all the source data underlying the results available to ensure full reproducibility?

Yes

Is the study design appropriate and is the work technically sound?

Yes

Are the conclusions drawn adequately supported by the results?

Yes

Are sufficient details of methods and analysis provided to allow replication by others?

Yes

Reviewer Expertise:

Macro-fungal taxonomy

I confirm that I have read this submission and believe that I have an appropriate level of expertise to confirm that it is of an acceptable scientific standard.

# Reviewer report: Reviewer response for version 1

**DOI:** 10.5256/f1000research.153750.r214959

**Published:** 2023-10-31

**Authors:** Nopparat Wannathes

**Affiliations:** 1 Microbiology Program, Faculty of Science and Technology, Pibulsongkram Rajabhat University, Phitsanulok, Phitsanulok, Thailand

The manuscript entitled "Morphological, phylogenetic, and genomic evidence reveals the causal agent of thread blight disease of cacao in Peru is a new species of *Marasmius* in the section Neosessiles, *Marasmius infestans* sp. nov." described a new species of *Marasmius* from Peru. This manuscript is interesting, with new data and nice photographs and drawings. However, some points need to be considered.

 This research reported a new species of *Marasmius*, which is a taxonomic report with some molecular evidence. So a clear morphological description of a new species is needed. The key characteristics of *Marasmius* species have to be informed, such as Melzer's reagent reactions of spores, and the trama tissues. A comparison of a new species with phenetically similar taxa is needed, especially for the species reported from the related geographical area --the Amazon rainforest, not scope to the *Marasmius* species that caused the thread blight disease (TBD) only. A comprehensive dataset for phylogenetics analyses is required, ITS sequences of related species from the Amazon rainforest (Oliveira *et al *2014--the sixth reference of this manuscript) have to be included. The phylogenetic analyses of a single gene of ITS and LSU and a combined gene of ITS and LSU are required at least as supplemented data. Due to the dataset used in this study being mainly based on both genes, Only a new species and a single specimen of undescribed *Marasmius* sp. have other genes. 

 There are some suggestions and comments, please look carefully at the following:

1. Introduction: the current classifications of *Marasmius* should be informed. Since, this research aimed to conduct a taxonomic and phylogenetic revision of the fungi in this genus. 
2. Methods: The spore Quotient (Q) should be determined, and the statistical analysis of spore sizes should show the arithmetic mean with standard deviations. The type specimens (holotype, lectotype, or neotype) are designated to a single specimen. So please correct the data in Table 1 and Figure 3. Please correct the abbreviation of the translation elongation factor 1-alpha gene. 
3. Results: Please make the scale bar in Figure 1 more clearly. In the Taxonomy subsection, the description that the author provided is not concordant with the specimen photo shown in Figure 1. Please correct the spacing, and number of the lamellae and clarify whether lamellae are intervernose or not. Please indicate the attachment of the stipe base and substrate. The Melzer's reaction is a taxonomic chemical reaction used in Marasmiaceae. Please inform the results of the test in the spores and all kinds of tissue. The color of spores, cells, and hypha are required to be stated in a description. The type of pileipellis, and all kinds of trama have to be informed. In Notes, a comprehensive comparison of related taxa is required especially for the Amazonia species.
4. Discussion: Since a new species reported in this study caused the TBD. The comparison of new species and other TBD causal species is valuable data. However, a species is described based on the sexual stage and the basidiocarp is mainly characterized. Other characters like the rhizomorph or culture morphology could be additional data. So please keep in mind that the TBD causal strains from West Africa are identified without the morphological characteristics of basidiocarp. The author cites that ITS did not provide sufficient support for resolving species in the section *Neosessiles* refers to the work of Wannathes et al 2009. I disagree with this information, please check this carefully. According to the number of available sequences in GenBank and the recent data on the barcode gaps in ITS of *Marasmius* (Wilson *et al., *2023 ^1^), it would be better if the author could investigate the phylogenetic analysis on combined sequences of ITS and LSU.

Is the work clearly and accurately presented and does it cite the current literature?

Partly

If applicable, is the statistical analysis and its interpretation appropriate?

Partly

Are all the source data underlying the results available to ensure full reproducibility?

No

Is the study design appropriate and is the work technically sound?

Partly

Are the conclusions drawn adequately supported by the results?

Partly

Are sufficient details of methods and analysis provided to allow replication by others?

Yes

Reviewer Expertise:

Taxonomy and systematics on agaric

I confirm that I have read this submission and believe that I have an appropriate level of expertise to confirm that it is of an acceptable scientific standard, however I have significant reservations, as outlined above.

## References

[1] : Does One Size Fit All? Variations in the DNA Barcode Gaps of Macrofungal Genera. *J Fungi (Basel)* .2023;9(8) : 10.3390/jof9080788 10.3390/jof9080788 37623559 PMC10455624

# Response

**Published:** 2024-01-18

**Authors:** Jorge Ronny Díaz-Valderrama

**Affiliations:** INDES-CES, Universidad Nacional Toribio Rodriguez de Mendoza de Amazonas, Chachapoyas, Amazonas, Peru

The manuscript entitled "Morphological, phylogenetic, and genomic evidence reveals the causal agent of thread blight disease of cacao in Peru is a new species of Marasmius in the section Neosessiles, Marasmius infestans sp. nov." described a new species of Marasmius from Peru. This manuscript is interesting, with new data and nice photographs and drawings. However, some points need to be considered.

 This research reported a new species of Marasmius, which is a taxonomic report with some molecular evidence. So a clear morphological description of a new species is needed. The key characteristics of Marasmius species have to be informed, such as Melzer's reagent reactions of spores, and the trama tissues. A comparison of a new species with phenetically similar taxa is needed, especially for the species reported from the related geographical area --the Amazon rainforest, not scope to the Marasmius species that caused the thread blight disease (TBD) only. A comprehensive dataset for phylogenetics analyses is required, ITS sequences of related species from the Amazon rainforest (Oliveira et al 2014--the sixth reference of this manuscript) have to be included. The phylogenetic analyses of a single gene of ITS and LSU and a combined gene of ITS and LSU are required at least as supplemented data. Due to the dataset used in this study being mainly based on both genes, Only a new species and a single specimen of undescribed Marasmius sp. have other genes.

  

- ***In this new version we have examined our specimens with Melzer’s reagent as suggested. ***
- ***Besides comparing the new species with TBD-causing Marasmius species, we have now included a discussion highlighting the main differences between the new species and phenetically similar taxa, such as M. neosessilis, M. griseoroseus, M. conchiformis, and M. jasingensis. ***
- ***In the phylogenetic analyses, we had included related species from Oliveira et al. (2014), such as Marasmius conchiformis and M. griseoroseus. In this new version, we are adding the other two species described in Oliveira et al. (2014), M. linderioides and M. longisetosus. In this regard, we are also including separate phylogenies with ITS, LSU, and concatenated data sets to accommodate reviewer suggestions. In all cases, phylogenies support that M. infestans is a new species.***

There are some suggestions and comments, please look carefully at the following:

 Introduction: the current classifications of Marasmius should be informed. Since, this research aimed to conduct a taxonomic and phylogenetic revision of the fungi in this genus.

***The current classification of Marasmius is now informed in the first paragraph of the introduction.***

 Methods: The spore Quotient (Q) should be determined, and the statistical analysis of spore sizes should show the arithmetic mean with standard deviations. The type specimens (holotype, lectotype, or neotype) are designated to a single specimen. So please correct the data in Table 1 and Figure 3. Please correct the abbreviation of the translation elongation factor 1-alpha gene.

- ***In this new version we have calculated the Quotient values of spores along with other important statistical metrics such as the arithmetic means and standard deviations, as suggested by the reviewer. ***
- ***We recognized the mistakes in the original submission regarding the designation of type specimens, as pointed out by the reviewer. Therefore, we have updated Table 1 and Figure 3 accordingly.***
- ***Regarding the abbreviation of the translation elongation factor 1-alpha gene, we have kept the abbreviation as “EF1α” following the nomenclature of Aime and Phillips-Mora (2005). We have also reviewed for consistency the abbreviations of other genes such as COX1 and updated the manuscript throughout.***

Results: Please make the scale bar in Figure 1 more clearly. In the Taxonomy subsection, the description that the author provided is not concordant with the specimen photo shown in Figure 1. Please correct the spacing, and number of the lamellae and clarify whether lamellae are intervernose or not. Please indicate the attachment of the stipe base and substrate. The Melzer's reaction is a taxonomic chemical reaction used in Marasmiaceae. Please inform the results of the test in the spores and all kinds of tissue. The color of spores, cells, and hypha are required to be stated in a description. The type of pileipellis, and all kinds of trama have to be informed. In Notes, a comprehensive comparison of related taxa is required especially for the Amazonia species.

- ***Scale bars have been adjusted for better clarity.***
- ***As pointed out by the reviewer, there were several inconsistencies and lack of details in the description section. We have made a thorough revision in this regard. We corrected the lamellae characteristics as follows: “Lamellae adnate, distant (1-5) with 0 series of lamellulae, narrow, non-reticulate, non-intervenose, white, non-marginate”. We have updated information about the stipe as follows: “Stipe insititious not arising from white rhizomorphs”. ***
- ***All microstructures were examined with Melzer’s reagent, finding that only Caulocystidia were strongly dextrinoid, while all other structures and tissues were inamyloid. We have included a new figure to show these findings.***
- ***The color of spores, cells, and other tissues was included in the description (hyaline, except for the Siccus-type broom cells that in some cases were light brown)***
- ***The type of pileipellis is hymeniderm, and other characteristics of trama tissues were added.***
- ***We have added a discussion comparing the new species with related taxa, with emphasis on Amazonian species from Oliveira et al. (2014).***

Discussion: Since a new species reported in this study caused the TBD. The comparison of new species and other TBD causal species is valuable data. However, a species is described based on the sexual stage and the basidiocarp is mainly characterized. Other characters like the rhizomorph or culture morphology could be additional data. So please keep in mind that the TBD causal strains from West Africa are identified without the morphological characteristics of basidiocarp. The author cites that ITS did not provide sufficient support for resolving species in the section Neosessiles refers to the work of Wannathes et al 2009. I disagree with this information, please check this carefully. According to the number of available sequences in GenBank and the recent data on the barcode gaps in ITS of Marasmius (Wilson et al., 20231), it would be better if the author could investigate the phylogenetic analysis on combined sequences of ITS and LSU.

***We have included a paragraph in which we have added a comparison of the new species with other related basidiocarp-forming species in the Neosessiles section. We agree with the reviewer, so we have clarified the statement in which we indicated that ITS did not provide sufficient phylogenetic resolution power within the Neosessiles. We have also added new phylogenies with ITS alone, LSU alone, and concatenated, to provide extra evidence for the new species.***

# Reviewer report: Reviewer response for version 1

**DOI:** 10.5256/f1000research.153750.r214961

**Published:** 2023-10-31

**Authors:** Atik Retnowati

**Affiliations:** 1 Herbarium Bogoriense, Cibinong, Indonesia

This is a study that aims to describe a new species of *Marasmius infestans* from Peru. Combination molecular and morphological study is the best approach to describe these new species. Overall, the work is good, but here are some suggestions:

- A mycobank registration number must be provided for a valid description.
- The previous report of *Marasmius* from Peru must be added to the introduction.
- Map of locality where the species was collected must be added.  It will help of the ordinary people to see where the exact type locality.
- Scale bar at the Figure 2 can clearly be seen, and it probably white color scale bar would be better.  
- Description: Lamellae adnate with 1-5 series of lamellulae.  It should be “Lamellae adnate, distant (1-5) with 0 series of lamellulae”.

Is the work clearly and accurately presented and does it cite the current literature?

Yes

If applicable, is the statistical analysis and its interpretation appropriate?

Yes

Are all the source data underlying the results available to ensure full reproducibility?

Yes

Is the study design appropriate and is the work technically sound?

Yes

Are the conclusions drawn adequately supported by the results?

Yes

Are sufficient details of methods and analysis provided to allow replication by others?

Yes

Reviewer Expertise:

Macro-fungal taxonomy

I confirm that I have read this submission and believe that I have an appropriate level of expertise to confirm that it is of an acceptable scientific standard, however I have significant reservations, as outlined above.

# Response

**Published:** 2024-01-18

**Authors:** Jorge Ronny Díaz-Valderrama

**Affiliations:** INDES-CES, Universidad Nacional Toribio Rodriguez de Mendoza de Amazonas, Chachapoyas, Amazonas, Peru

This is a study that aims to describe a new species of Marasmius infestans from Peru. Combination molecular and morphological study is the best approach to describe these new species. Overall, the work is good, but here are some suggestions:

 A mycobank registration number must be provided for a valid description.

***To register a new fungal name, you need to do it in only one recognized repository (Aime et al., 2021). The recognized repositories for fungal names are: Fungal Names, Index Fungorum, and MycoBank (Aime et al., 2021). We have registered “Marasmius infestans” in Index Fungorum (IF 901138), so there is no need for a MycoBank registration number.***

***- Aime, M. C., Miller, A. N., Aoki, T., Bensch, K., Cai, L., Crous, P. W., ... & Schoch, C. L. (2021). How to publish a new fungal species, or name, version 3.0. IMA fungus, 12, 1-15.***

 The previous report of Marasmius from Peru must be added to the introduction.

***The report of the thread-blight disease-causing Marasmius species in Peru (Huamán et al., 2023; reference number 7 in the manuscript) is in the introduction as follows:***

***“…*** ***in native Awajun and Wampis communities from Northern Peru ^7^”***

 Map of locality where the species was collected must be added.  It will help of the ordinary people to see where the exact type locality.

***A map is now included in version 2 of the manuscript as Figure 1. Because of this, all other figure numbers have been updated.***

 Scale bar at the Figure 2 can clearly be seen, and it probably white color scale bar would be better. 

***We have made the scale bars wider and of white color, so they are more visible to the reader.***

 Description: Lamellae adnate with 1-5 series of lamellulae.  It should be “Lamellae adnate, distant (1-5) with 0 series of lamellulae”.

***We agree with this comment and the statement was updated as indicated.***
